# Design of transfections: Implementation of design of experiments for cell transfection fine tuning

**DOI:** 10.1002/bit.27918

**Published:** 2021-09-01

**Authors:** Sara Mancinelli, Andrea Turcato, Annamaria Kisslinger, Antonella Bongiovanni, Valeria Zazzu, Antonella Lanati, Giovanna Lucia Liguori

**Affiliations:** ^1^ Institute of Genetics and Biophysics (IGB) National Research Council (CNR) Naples Italy; ^2^ Valore Qualità Pavia Italy; ^3^ Institute of Experimental Endocrinology and Oncology (IEOS) National Research Council (CNR) Naples Italy; ^4^ Institute for Research and Biomedical Innovation (IRIB) National Research Council (CNR) Palermo Italy; ^5^ Present address: Sara Mancinelli, Department of Biomedical Sciences Humanitas University, Via Rita Levi Montalcini 4, 20090 Pieve Emanuele, Milan, Italy and IRCCS Humanitas Research Hospital Via Manzoni 56 Rozzano Milan 20089 Italy

**Keywords:** cell transfection, design of experiments (DoE), neural progenitors, optimization, polyethyleneimine (PEI)

## Abstract

Transfection is the process by which nucleic acids are introduced into eukaryotic cells. This is fundamental in basic research for studying gene function and modulation of gene expression as well as for many bioprocesses in the manufacturing of clinical‐grade recombinant biologics from cells. Transfection efficiency is a critical parameter to increase biologics' productivity; the right protocol has to be identified to ensure high transfection efficiency and therefore high product yield. Design of experiments (DoE) is a mathematical method that has become a key tool in bioprocess development. Based on the DoE method, we developed an operational flow that we called “Design of Transfections” (DoT) for specific transfection modeling and identification of the optimal transfection conditions. As a proof of principle, we applied the DoT workflow to optimize a cell transfection chemical protocol for neural progenitors, using polyethyleneimine (PEI). We simultaneously varied key influencing factors, namely concentration and type of PEI, DNA concentration, and cell density. The transfection efficiency was measured by fluorescence imaging followed by automatic counting of the green fluorescent transfected cells. Taking advantage of the DoT workflow, we developed a new simple, efficient, and economically advantageous PEI transfection protocol through which we were able to obtain a transfection efficiency of 34%.

## Graphical abstract

Taking advantage of the Design of Experiment method, we developed a flexible operational workflow that we called “Design of Transfections” (DoT), aimed at obtaining an effective, standardized, and reproducible cell transfection procedure, suitable for different cell types and transfection reagents. The application of the DoT workflow let us to develop a new, simple, efficient, and economically advantageous transfection protocol for neural progenitors using linear polyethyleneimine (PEI).

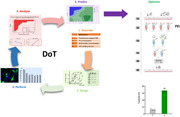

## 1 INTRODUCTION

The transfection process is extremely useful in basic research to study the role of a specific gene through experiments of both gain of function (DNA transfection) or loss of function (small interfering RNA transfection), as well as for modulation of gene expression, mutational analysis and recombinant protein production (Kim & Eberwine, 2010). Moreover, transfection is fundamental in several therapeutic approaches based on gene delivery strategies (Neshat et al., 2020; Pfeifer & Verma, 2001), for the genetic modification of cells of human origin, for example, for the generation and engineering of the induced pluripotent stem (iPS) cells to be used as models of disease and/or in patient‐specific cell therapy (Nishikawa et al., 2008; Shankar et al., 2020; Takahashi & Yamanaka, 2006) as well as in many bioprocesses for the production of clinical‐grade recombinant biologics (Wurm, 2004).

Cultivated mammalian cells have become the dominant system for the production of recombinant proteins for clinical applications because of their capacity for proper protein folding, assembly, and posttranslational modifications. Biotherapeutics are generally secreted by genetically engineered mammalian cell lines into their culture medium, they can then be purified to homogeneity and sterilized under well‐defined and regulated conditions. In 1986, human tissue plasminogen activator (tPA, Activase; Genentech) became the first therapeutic protein from recombinant mammalian cells to obtain market approval. Since then, monoclonal antibodies, fusion proteins, and enzymes have been produced to treat various pathologies such as cancer, and metabolic and autoimmune diseases (Wurm, 2004). Their successes have driven substantial increases in clinical trials over the years, resulting in a steady growth of the number of recombinant protein therapeutics on the market, with over 100 products approved by the US FDA in 2015, most of which were produced by genetically engineered mammalian cells (Kinch, 2016). Recently, the productivity of mammalian cells cultivated in bioreactors has been greatly enhanced through improvements in media composition and process control. A great deal of effort has been applied to the establishment of methods for efficient product scale‐up and cost reduction of manufacturing processes. The development of a manufacturing process for a recombinant protein in mammalian cells follows a well‐established scheme starting with the transfer of the recombinant gene with the necessary transcriptional regulatory elements to the cells by transfection (Wurm, 2004). Transfection efficiency is a key parameter to increase biologics' productivity. Many transfection methods have been developed, broadly classified into biological (mediated by viruses, virus‐like particles, or extracellular vesicles), physical (such as electroporation, magnetofection, sonoporation biolistic particle delivery, and laser‐mediated transfection) and chemical methods, each one showing particular characteristics, benefits, and disadvantages (Chong et al., 2021; Fajrial et al., 2020; Gouvarchin Ghaleh et al., 2020; Harris & Elmer, 2020; Kim & Eberwine, 2010; Mantile et al., 2020; Mykhaylyk et al., 2007; Sarvaria et al., 2017; Scherer et al., 2002; Schillinger et al., 2005; Slivac et al., 2017).

Chemical transfection methods were the first to be used to introduce foreign genes into mammalian cells and are still the most widely used methods. The underlying principle of chemical transfection relies on the formation of a complex between positively charged chemicals (calcium phosphate, polymers, and lipids) and negatively charged nucleic acids. Such complexes are subsequently attracted to the negatively charged cell membrane and pass through it, probably through mechanisms involving endocytosis and phagocytosis. Compared with the nonchemical ones, chemical methods have the merits of relatively low cytotoxicity, ease of use, cost‐effectiveness, with no mutagenesis, no viral vector involvement, no size limitation on the packaged nucleic acid, and no safety problems (Kim & Eberwine, 2010). The Achilles' heel of chemical transfection is the transfection efficiency, it being lower than in biological methods based on viral vectors and also highly variable in response to different factors. Depending on the cell type and the molecule to transfect, the correct protocol has to be identified to maximize transfection efficiency and product yield.

Traditional experimentation in cell biology, as elsewhere, has typically been conducted using a one‐factor‐at‐a‐time (OFAT) approach, in which every factor (variable) is kept constant except for the factor under investigation that is varied with the resulting output being measured. However, the complexity of cell processes requires the simultaneous examination of several input variables that must be controlled. Design of experiments (DoE) is a mathematical method for planning, conducting, analyzing, and interpreting controlled tests of multivariable processes. DoE allows to identify the factors that significantly influence the desired output, their interactions, and ultimately the best combinations of factors that maximize the output and optimize the process (Montgomery, 2012; Myers et al., 2016). DoE has been widely used to maximize yields and improve processes at an early stage, leading to major benefits in both product performance as well as management of resources (Grangeia et al., 2020; Mandenius & Brundin, 2008; Politis et al., 2017; Weissman & Anderson, 2015). In recent years, the use of the DoE approach as an alternative to the traditional OFAT method is definitely increasing in many fields of scientific research, including cell biology, biochemistry, and nanotechnologies, to identify the main factors controlling the scientific process and for modeling their effects (Bollin et al., 2011; Durakovic, 2017; Esteban et al., 2021; Lanati, 2018; Mancinelli et al., 2015; Narenderan et al., 2019; Papaneophytou et al., 2021; Papaneophytou, 2019; Tavares Luiz et al., 2021; Toms et al., 2017; Xu et al., 2020). In this scenario, we have been committed for many years in developing, applying, and validating models that can be useful for the management or research activities, helping in standardizing, and optimizing processes, thus improving data reliability and reproducibility (Bongiovanni et al., 2015; Digilio et al., 2016; Liguori & Kisslinger, 2020; Mancinelli et al., 2015; Mascia et al., 2020).

In the present study, we focused on the application of DoE to the analysis, modeling, and optimization of a cell transfection protocol. Previous DoE applications to cell transfection mainly focused on CHO or HEK cell lines, although they differed for the factors analyzed, the methodological approach proposed, the transfection output selected, and the relative measurements, ranging from the titer and quality of the produced antibody to fluo‐cytometer or fluorimetric analysis of GFP expression (Agirre et al., 2015; Bollin et al., 2011; Cervera et al., 2015; Elshereef et al., 2019; Thompson et al., 2012). Ultimately, the outcome of these studies pointed to the suitability of cell transfection for DoE application in different contexts. As far as we could ascertain, our study applies for the first time the DoE method to noncommercial cell lines, specifically neural progenitor cells that are widely recognized as difficult to transfect (Alabdullah et al., 2019; Karra & Dahm, 2010). We choose to test polyethyleneimine (PEI) as a transfection reagent for its ease of use and its relatively low costs (Neuberg & Kichler, 2014) and to analyze the effect of PEI type, PEI concentration, DNA concentration, and cell density on cell transfection efficiency as measured by fluorescence imaging. We clearly defined a sequential approach, using first a two‐level full factorial design to study the effect of each factor on transfection and all the possible factor interactions. Second, a response surface methodology was applied to identify the best factor combinations that optimize transfection as well as to develop a predicting model describing the relation between transfection efficiency and its most influential factors. This flexible operational flow, aimed at obtaining an effective, standardized, and reproducible cell transfection procedure, suitable for different cell types and transfection reagents, we called “Design of Transfections” (DoT).

## 2 MATERIALS AND METHODS

### 2.1 Cell culture and transfection

**Figure 1:**
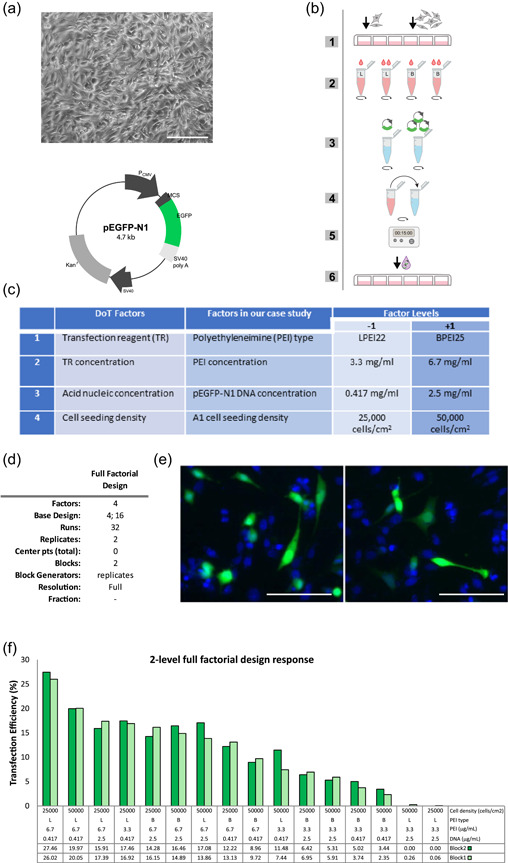
Transfection setting and factorial design. (a) mes‐c‐myc A1 cell culture and map of the pEGFP‐N1 plasmid. (b) Schematic representation of the cell transfection protocol through sequential steps: (1) cell seeding, (2) PEI dilution, (3) DNA dilution, (4) DNA‐PEI mixing, (5), incubation and DNA‐PEI complex formation, and (6) exposure of the DNA‐PEI complexes to cells. (c) List of the four factors identified for the design of transfections (DoT), the specific factors analyzed in the factorial design, and the corresponding high (+1) and low (−1) levels chosen. (d) Main features of the full factorial design. (e) Representative images of the results of cell transfection under fluorescence microscopy. (f) Bar plot showing the transfection efficiency obtained for the 16 different combinations analyzed in replicates (Blocks 1 and 2). Scale bars indicate 100 μm in (a) and 50 μm in (e)

Mes‐c‐myc A1 (A1) cells are noncommercial immortalized progenitors derived from mesencephalon of mouse embryos at 11 days of development and infected with a replication‐defective retrovirus bearing c‐myc, expressing both markers of neural precursors as well as neuronal markers (Colucci‐D'amato et al., 1999). A1 cells (Figure 1a) were cultured in Minimum Essential Medium (Gibco™) and F12 medium (Gibco™) 1:1 (vol/vol) supplemented with 10% fetal bovine serum (Gibco™). In these conditions, the cells proliferate and maintain all the characteristics of neural progenitors (Colucci‐D'amato et al., 1999). The plasmid chosen for transfection is pEGFP‐N1 (Clontech) carrying the coding sequence for green fluorescence protein (GFP) downstream to the promoter of cytomegalovirus (Figure 1a). To set the transfection protocol, we used as reference the protocol developed by Ming Hsu and UludaĞ (2012) to efficiently transfect primary tissue‐derived cells (fibroblasts and bone marrow stromal cells) using PEI (Ming Hsu & UludaĞ, 2012), and adapted it to our cellular system (Figure 1b). We tested both 25 kDa branched (BPEI25) and 22 kDa linear PEI (LPEI22). Twenty‐four hours before transfection, A1 cells were seeded in 24‐well plates with 0.5 ml of medium/well at the densities fixed in the experimental design. Complexation between DNA and PEI was performed through a two‐part mixing in culture medium. Moreover, for each factor analyzed, we tested different levels according to the DoE approach. According to the experimental design, different volumes of PEI, linear or branched, (1 μg/ml, pH 7.0) (SIGMA), were added in a 1.5‐ml tube to antibiotic‐ and serum‐free culture medium to a final volume of 50 μl. In a second tube, the DNA volumes indicated by the experimental design were added to antibiotic‐ and serum‐free culture medium to a final volume of 50 μl as well. The two solutions were vortexed and incubated for 10 min at room temperature (RT) to allow DNA and PEI to dissolve properly in the medium, then were mixed together. The resulting transfection solution (final volume of 100 μl) was vortexed and incubated 15 min at RT to allow the formation of PEI‐DNA complexes and then was added drop by drop to the A1 cell culture, and left in the cell incubator at 37°C in 5% CO_2_ and 20% O_2_. The transfection medium was replaced after 16 h of incubation with normal culture medium.

### 2.2 CellProfiler pipeline for transfection efficiency computation

Twenty‐four hours after transfection, cells were fixed in 4% paraformaldehyde for 15 min at RT, and Hoechst (Thermo Fisher Scientific) counterstained following the manufacturer's instructions. Cells transfected with pEGFP‐N1 plasmid acquired the ability to express GFP, and could then be visualized by means of fluorescence microscopy. Ten randomly chosen areas for each design run were captured as images and further processed using a specifically generated pipeline and the CellProfiler image analysis software. Single‐channel 8‐bit images were uploaded in the proper section of the CellProfiler software and a unique text pattern was chosen to identify each channel (e.g., green channel image = ch01; blue channel image = ch02). The identification of both Hoechst counterstained nuclei (in blue) and GFP positive cells (in green) was obtained through an unbiased segmentation algorithm and generation of a biunivocal relation between nuclei (parent objects) and GFP positive cells (child objects) to filter transfected cells. The percentage of transfected cells was calculated as the ratio between the number of child objects over the number of parent objects.

### 2.3 Experimental design

First of all, factors to be tested for their effect on transfection efficiency were identified together with a suitable value range. The designs of the experiments were then generated through Minitab Statistical Software version 16 and version 19 (www.minitab.com; Minitab Inc.), following suggestions by the Quality Companion 3 by Minitab. The first experimental design chosen for this study was a two‐level full factorial design, including 2^*k*^ different combinations (where *k* represents the number of factors analyzed) and two possible values or levels, high (+1) and low (−1) for each factor (Montgomery, 2012; Myers et al., 2016). With four factors, the design included 2^4^ = 16 different combinations, that became 32, since we chose to perform the tests in duplicate. We then performed a successive protocol optimization by using the following response surface designs: (i) the Box–Behnken design (BBD), which includes a number of combinations to tests *N* = 2 *k*(*k* − 1) + C_0_, where *k* is the number of factors, and C_0_ is the number of central points (Ferreira et al., 2007) and (ii) a Box–Wilson central composite design (CCD), which consists of a two‐level (–1 and + 1) factorial, augmented with further points (axial or star points and central points), allowing the estimation of pure quadratic effects (Myers et al., 2016; Tavares Luiz et al., 2021). We first tested by BBD three factors and 15 combinations and finally by CCD two factors and 11 combinations, setting an alpha value of 1.41, where alpha is the distance of each axial point from the center of the design space. Even for BBD and CCD, combinations were tested with replicates (meaning 30 total experimental runs for BBD and 22 for CCD) identified by separate blocks to account for possible differences in experimental conditions. All the experiments were performed according to the experimental design generated by Minitab software. The transfection efficiency of each run was measured as described above and the data produced, the main factor effects and their interactions were analyzed through Minitab statistical software.

### 2.4 Modeling and validation

The data obtained from the full factorial and both the response surface designs, keeping constant the optimized levels of PEI type and cell density, were combined and analyzed together by using MiniTab statistical software to generate a response surface plot and a contour plot. The plots describe how the combination of the influential factors affects transfection efficiency (the response output) and allow us to identify the setting of factors that maximizes the output. To validate the model, we tested, in triplicates, two different factor settings, corresponding to the optimal conditions found (6.5 μg/ml LPEI22, 1 μg/ml DNA, 25,000 cells/cm^2^) and a suboptimal one (5 μg/ml LPEI22, 2 μg/ml DNA, 25000 cells/cm^2^). The protocol used was the one described above. Two other contour plots reporting the transfection efficiency with respect to PEI/DNA ratio and DNA or PEI respectively were generated to identify the best ratio range.

## 3 RESULTS AND DISCUSSION

### 3.1 Experimental setting

Chemical transfection efficiency varies depending on cell type, genetic material to be introduced, and chemical method adopted, and is largely affected by several parameters, both quantitative and qualitative (Kim & Eberwine, 2010). Here, we present a DoT operational flow to identify the parameters that significantly affect transfection, and, then, to identify the best setting which maximizes transfection efficiency.

As proof of concept, we chose to transfect cells of neural origin that are widely recognized as extremely difficult to transfect and with a low response to traditional lipidic transfection methods (Alabdullah et al., 2019; Karra & Dahm, 2010). Specifically, we transfected the A1 cells (Colucci‐D'amato et al., 1999) with a DNA vector containing a sequence encoding GFP, that allowed a simple and automatic identification and counting of the transfected cells through an “ad‐hoc” pipeline, ensuring greater reproducibility, accuracy, and speed than manual counting. Once the cell line, the nucleic acid to transfect, and the reference protocol were fixed, the transfection variables to analyze and the possible values or “levels” for each factor were identified. Not only the choice of the factors to be tested, but also the choice of the relative levels is really crucial: a narrow interval between levels might prevent a full analysis of the whole frame of combinations, whereas a large interval increases the probability that nonlinear effects may confound the analysis. In some cases, preliminary experiments are advisable to identify the most promising factors as well as the extremes of the interval to analyze, avoiding affecting cell viability, for instance. In our DoT assay, we chose to test the four factors listed in Figure 1c.

#### 3.1.1 Factor 1 (qualitative): type of chemical transfection reagent

As a chemical method, we choose PEI, a cationic nonlipidic transfection reagent normally used to achieve higher transfection efficiencies in cell lines that are refractory to liposome‐based transfection (Ming Hsu & UludaĞ, 2012). PEI has also been recently used associated to nanoparticles in physical transfection methods based on magnetofection (Cen et al., 2019; Song et al., 2019). Even though lipofection is considered the “gold‐standard” to which other techniques are usually benchmarked, the quantitative evaluation of transfection rates for cells of neural origin using Lipofectamine 2000 showed poor efficiency, recently estimated at precisely 10%–12% for neuroblastoma cells, 5%–12% for primary astrocytes and only 1.3%–6% for primary neurons (Alabdullah et al., 2019). For this reason, we decided to test PEI as an alternative method. PEI comes in linear and branched configurations, ranging from low to high‐molecular weight. The transfection efficiency of PEI is closely tied to nucleic acid binding and dissociation of the polymer. Low‐molecular‐weight PEIs have fewer amine groups per molecule, which bind and condense DNA less efficiently, resulting in lower overall transfection efficiencies. By contrast, PEI molecules of high molecular weight have a less effective release, also leading to reduced transfection efficiency, in addition, they are more toxic, reducing the viability of cells for transgene expression. Therefore, midrange molecular‐weight PEIs, such as BPEI25 and LPEI22, provide a balance between binding affinity and ease of dissociation and are the most popular and effective transfection agents. By using PEI‐based protocols a transfection efficiency of around 30%–35% was obtained for normal human foreskin fibroblasts and of 8%–12% for bone marrow stromal cells (Ming Hsu & UludaĞ, 2012), whereas the optimization of neural cells transfection by means of PEI molecules has not yet been specifically reported.

#### 3.1.2 Factors 2 and 3 (quantitative): concentration of transfection reagent (2) and concentration of nucleic acid (3)

It is widely considered that the higher the concentration of nucleic acid administered to the cells, the higher is the level of transfection. However, the amount of DNA that can be applied in transfection is limited by the final concentration of polymer and the ratio of polymer to DNA. An excess of polymer is essential for intracellular trafficking and for overcoming the inhibitory effect of the anionic cell‐surface glycosaminoglycan (GAG). However, if the amount of polymer becomes too high, it causes toxicity and reduces overall viability. As cell physiology affects a range of metabolic activities, including uptake pathway and the expression of cell surface receptors, the amount of surface GAGs is likely to differ between cell lines. Thus, the optimal polymer and, consequently nucleic acid concentration, needs to be empirically optimized for each cell line (Ming Hsu & UludaĞ, 2012). The concentration of PEI to test was chosen taking into account previous DoE‐based analysis of the effect of PEI on A1 cell viability identifying a negative correlation between cell viability and PEI amount/cell (Mancinelli et al., 2015). The high PEI concentration level was fixed at 6.7 μg/ml, corresponding to a PEI amount/cell ranging between 17.63 and 35.26 pg and cell viability ranging approximately between 56% and 75%. The DNA concentration was chosen accordingly to analyze polymer‐to‐DNA weight ratio levels ranging from 1.32 to 16.07, in agreement with PEI/DNA weight ratios analyzed in the PEI transfection protocol optimized by Ming Hsu and UludaĞ (2012) through the OFAT method.

#### 3.1.3 Factor 4 (quantitative): cell density

The density of cells during transfection is closely linked to the polymer and DNA concentrations. If cell density is low, the concentration of polymer would be relatively high compared with transfection in high‐density cell culture and might affect cell viability (Mancinelli et al., 2015; Ming Hsu & UludaĞ, 2012). As in many transfection protocols, we chose to refer to the seeding density, which can be measured more precisely, even though it might not necessarily resemble the attached cell density, depending on culturing conditions, handling processes, and age of cell culture. The cell density levels chosen were the ones for which A1 cells were shown to be in a logarithmic phase of proliferation, fundamental for a good DNA uptake efficiency (Mancinelli et al., 2015).

### 3.2 Factorial design and identification of significant factors and interactions

**Figure 2:**
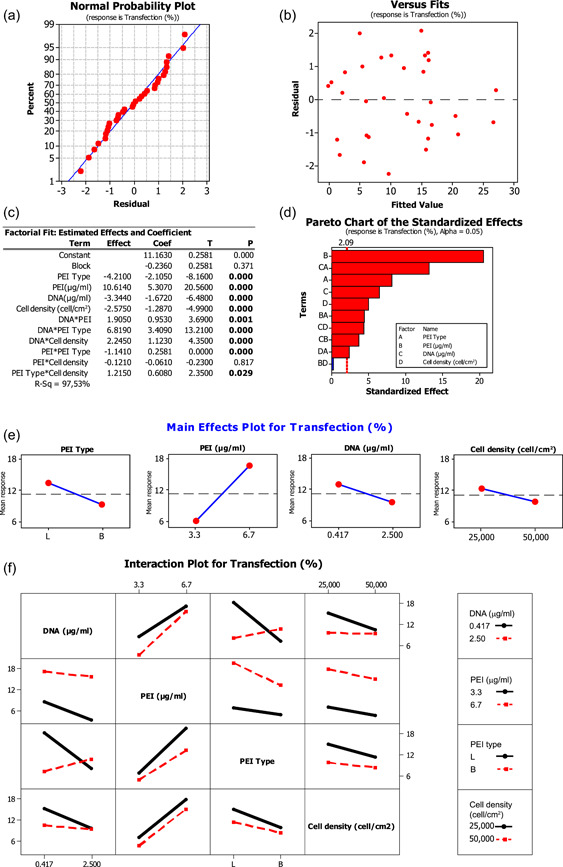
Residual, effect, and interaction analysis of the full factorial design. (a) Normal probability plot showing that residual distribution fitted with a normal distribution. (b) Scattered dot plot of residuals compared to fitted values showing constant variance. (c) Factorial fit table and (d) Pareto chart of the standardized effects. (e) Main effects plots showing the mean transfection efficiency response at the different factor levels. (f) Interaction plots reporting how significant two‐factor interactions affected transfection efficiency at the different factor levels

Once we chose the factors to test, we first performed a full factorial experiment. The full factorial design consists of two or more factors, each with discrete levels, whose experimental runs adopt all possible combinations of these levels across all such factors. Such a design allows users to analyze the effect on the response output of each factor as well as of the interactions between them (Montgomery, 2012; Myers et al., 2016). Due to its experimental simplicity coupled with improved statistical efficiency, we performed a two‐level full factorial design with four factors on two levels each (−1; +1) that were: (i) PEI type (L = linear; and B = branched); (ii) PEI concentration (3.3 and 6.7 μg/ml); (iii) DNA concentration (0.471 and 2.5 μg/ml); and (iv) cell density (25,000 and 50,000 cells/cm^2^) as summarized in Figure 1c. With four factors, the design included 16 different combinations, which became 32 as we chose to perform the tests in duplicate. The worksheet generated by Minitab Statistical software is shown in Supplementary Table, whereas all the features of the design chosen are reported in Figure 1d. Because the two replicas were performed at two different times of the day, two blocks were used to take into account possible variability not due to the analyzed factors. The output variable, that is the transfection efficiency of each test, was calculated as the number of transfected green fluorescent cells, expressing GFP, on the total number of cells, visible as blue after Hoechst counterstaining of nuclei (Figure 1e). The transfection efficiency obtained varied between 0% and 27.46%, as reported in the bar plot in Figure 1f, denoting that the factorial design explored a significant and informative interval of possible combinations. The data were analyzed by MiniTab software, confirming a normal distribution and constant variance of the residuals (Figure 2a,b). As shown in the factorial‐fit table (Figure 2c), the *p* value for the block parameter was not significant (*p* = 0.371), meaning that the obtained results in the two replicas were not influenced by external factors, such as replication‐related noise, daytime, different times of execution or other variables not modeled in the experimental design. Moreover, both the factorial‐fit table (Figure 2c) and the Pareto chart of the standardized effects (Figure 2d) showed that all the four factors tested had a significant effect on transfection (Figure 2c). The most influential factor was by far the concentration of PEI (effect = 10.61, *p* < 0.0001), followed in order by the type of PEI (effect = −4.21, *p* < 0.0001), the DNA concentration (effect = −3.34, *p* < 0.001), and the cell seeding density (effect = −2.57, *p* < 0.001). As shown in the main effect plots (Figure 2e), the higher mean transfection efficiency (MTE) corresponded for PEI concentration at the high level (6.7 μg/ml, MTE = 16.47%), whereas it corresponded to the low level for both DNA concentration (0.471 μg/ml, MTE = 12.84%) and cell density (25000 cells/cm^2^, MTE = 12.45%). Interestingly, the LPEI22 polymer was more suitable for A1 cell transfection than the BPEI25 (13.21% MTE against 9.06%) that was used to efficiently transfect primary tissue‐derived cells of different origin (Ming Hsu & UludaĞ, 2012), confirming how optimal experimental conditions may significantly differ from one type of cells to another. Moreover, all these data indicated that the factors, as well as the levels chosen, were good options to test in the DoT assay.

A more complete scenario arose when analyzing the significant two‐way factor interactions (Figure 2c,d,f). In the analyzed interval, five interactions were significant: DNA concentration × PEI type (effect = 6.82, *p* < 0.0001); DNA concentration × cell density (effect = 2.24, *p* < 0.0001); DNA concentration × PEI concentration (effect = 1.90, *p* = 0.001); PEI type × cell density (effect = 1.21, *p* = 0.029) and PEI concentration × PEI type (effect = −1.14, *p* < 0.0001). The only one resulting not significant in the analyzed factor interval was the interaction between PEI concentration and cell density (effect = −0.12, *p* = 0.817), which actually had been already optimized in previous experiments to avoid high cytotoxic effect of PEI (Mancinelli et al., 2015). Among the significant interactions, the most interesting one was the interaction between PEI type and DNA concentration. Even though the best combination is LPEI22 with the low level of DNA concentration (0.417 μg/ml), corresponding to 18.35%, of MTE, interestingly, at the high level of DNA concentration (2.5 μg/ml) it is the BPEI25 that acts more efficiently (MTE = 10.80%), as shown in Figure 2f. The interaction between DNA and PEI concentration is also relevant, as transfection efficiency at the low level of PEI concentration is more influenced by DNA concentration (Figure 2f). Our data confirmed that the cell transfection by means of cationic polymers is strongly affected by the equilibrium between the number of negative DNA charges and the number of positive charges on the polymer, which are indeed fewer on the linear than on the branched PEI molecules. In addition, PEI type significantly influenced the effect on transfection of both PEI concentration and cell density. The high level of PEI concentration corresponded in all combinations to higher overall transfection efficiency, independently of its conformation (16.47% MTE at 6.7 μg/ml against 5.80% at 3.3 μg/ml), as shown in Figure 2e. However, LPEI22 led to the best transfection output compared to BPEI25 at both high (19.72% MTE for LPEI22 against 13.22% for BPEI25) and low (6.70% MTE for LPEI22 against 4.89% for BPEI25) PEI concentration levels (Figure 2f) as well as at both cell densities tested (15.15% MTE for LPEI22 against 9.74% for BPEI25 at 25,000 cells/cm^2^; 11.27% MTE for LPEI22 against 8.38% for BPEI25 at 50,000 cells/cm^2^). Finally, the three‐way interaction among the type of PEI, cell density, and DNA concentration was also found significant (*p* = 0.014; data not shown), confirming that the optimal ratio between PEI positive charges and DNA negative charges for cell transfection changes depending also on cell seeding density.

The interaction analysis clearly showed how the traditional OFAT approach, which does not take into account all the possible interactions among factors, might severely limit the optimization of a multivariable assay, possibly leading to the identification of a relative maximum output thus hiding the potential best achievable one. Overall, the main effect and interaction analysis ultimately determined LPEI22 as the PEI conformation to use with A1 cells.

### 3.3 Optimization design

Once fixed LPEI22 as the PEI conformation to use, PEI concentration, DNA concentration, and cell density, all significant with respect to the output, were analyzed in the subsequent optimization design to obtain a model describing the transfection efficiency variability as a combination of interacting significant factors. For the optimization, we took advantage of the response surface designs, mainly the BBD and the CCD, used to identify the points of absolute maximum and to highlight possible nonlinearities, by adding points to the factor space analyzed (Lanati, 2018; Myers et al., 2016). The addition of more points to the factor space strengthened the analysis and let us model more precisely the relationship among the output variable and the input factors.

**Figure 3:**
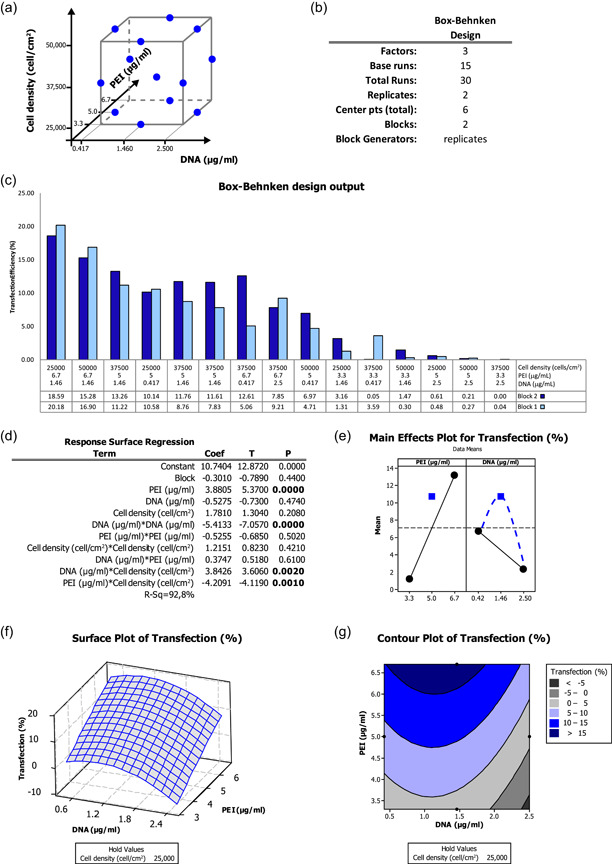
Analysis of the results of the Box–Behnken design. (a) Schematic representation of the points analyzed. (b) Main features of the design. (c) Bar plot of the transfection efficiency obtained for the 15 different combinations in replicates (Blocks 1 and 2). (d) Response surface regression showing that PEI concentration and DNA concentration square term are significant. (e) Main effects plot highlighting a curvature in the relation between transfection efficiency and DNA concentration. (f) Surface and (g) contour plots showing how transfection efficiency changes depending on PEI and DNA concentration in the interval analyzed

We first ran a BBD, which for three factors requires fewer combinations than the CCD. The BBD takes into account the midpoints of edges of the process space as well as the center levels (Figure 3a), and, for a three factors' analysis, includes 15 runs (instead of the 20 required for CCD), becoming 30 with replicates, as summarized in Figure 3b. All the combinations analyzed and the relative transfection efficiencies are reported in the bar plot in Figure 3c and detailed in Figure S1a. The statistical analysis showed normal distribution and variance of the residuals (Figure S1B). Noteworthy, following the analysis of the response surface regression (Figure 3d), the square term of the DNA concentration showed significance (*p* < 0.00001), indicating the presence of a statistically significant curvature in the main effect of this factor on the transfection output. Conversely, the square term related to PEI concentration was not significant (*p* = 0.502), indicating that the relation between PEI concentration and transfection efficiency is linear within this interval (Figure 5d). These data were clearly shown in the main effect plot in Figure 3e, highlighting for DNA concentration (but not PEI concentration) a significant deviation of the effect at the center point (MTE = 10.12%), with respect to the average mean response of the factors at their low and high levels, that is 6.71% and 2.33%, respectively (Figure 3e). Moreover, with the addition of the midpoints, cell density, whose standardized effect was the least significant in the factorial experiment (Figure 2c,d), no longer showed a significant effect on the experimental output (*p* = 0.421, Figure 3d). Therefore, this factor did not require any additional optimization and was set constant at the lower level of 25,000 cells/cm^2^ to save time and experimental material. The variation of the transfection output depending on the simultaneous variation of both PEI and DNA concentration (keeping cell density at 25,000 cells/cm^2^) was reported in both a three‐dimensional response surface plot (Figure 3f) and a two‐dimensional contour plot (Figure 3g). The contour plot highlighted a response surface “rising ridge,” with the best output lying at the edge of the plot at the high level of PEI concentration and centered mainly in the lower half of the analyzed DNA concentration range.

**Figure 4:**
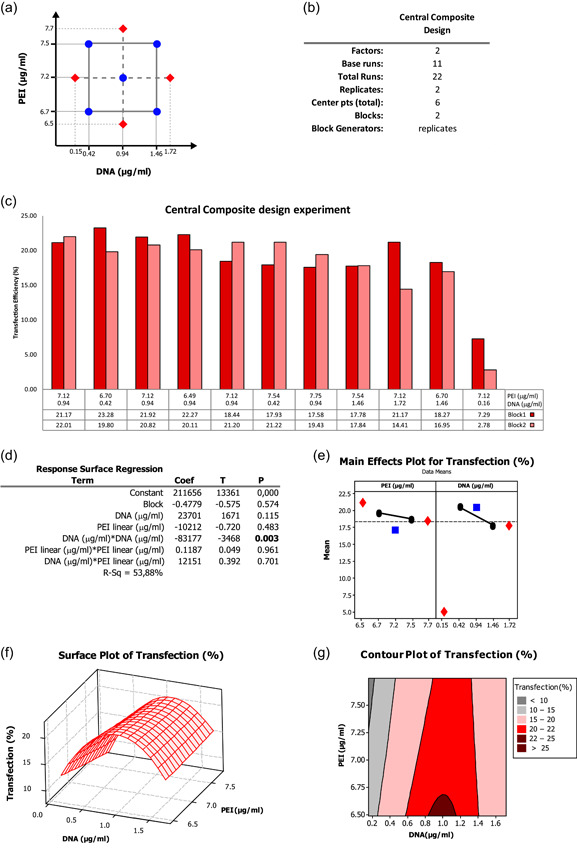
Analysis of the results of the central composite design. (a) Schematic representation of the points analyzed. (b) Main features of the design. (c) Bar plot of the transfection efficiency obtained in the 11 different combinations in replicates (Blocks 1 and 2). (d) Response surface regression showing the significance of the DNA concentration square term. (e) Main effects plot. Red points indicate the transfection efficiency obtained at the axial point factor levels while blue ones correspond to the center point factor levels. (f) Surface and (g) contour plots showing how transfection efficiency changes depending on PEI and DNA concentration in the space analyzed

As a further step, we refined the analysis by focusing on lower levels of DNA concentration and slightly higher levels of PEI concentration (up to 7.75 μg/ml), although corresponding to a decreased cell viability moving close to 50% (Mancinelli et al., 2015). The other two factors, PEI type and cell density were kept constant. With this aim, we took advantage of a CCD, centered on the most promising area of the factor space, to provide high‐quality predictions by adding more points and above all by exploring also factor levels outside the reference interval investigated in the previous designs (Figure S2). The CCD includes star points that are fixed at a distance from the design center identified by the α parameter, set at the standard value of 1.41 (Figure 4a). CCD main features for two factors and five levels are schematized in Figure 4b. The combinations identified by the design together with the transfection efficiencies obtained are graphically displayed in the bar plot in Figure 4c and detailed in Figure S3a. The statistical analysis assessed the normal distribution and variance of the residuals (Figure S3b). The analysis of variance of this new experimental set confirmed the significance of the DNA concentration square term (*p* = 0.008), while the addition of the star points let us better model the curvature for this factor (Figure 4d,e). Regarding the PEI concentration, the CCD, as the previously run BBD, did not detect any significant curvature (*p* = 0.483, Figure 4d,e). The resulting response surface plot (Figure 4f) and contour plot (Figure 4g) outlined a response surface, showing an output maximum at approximately 1 μg/ml of DNA and demonstrating that increased PEI concentrations did not improve, rather worsened the output, probably also due to their cytotoxic effects.

### 3.4 Modeling and validation

**Figure 5:**
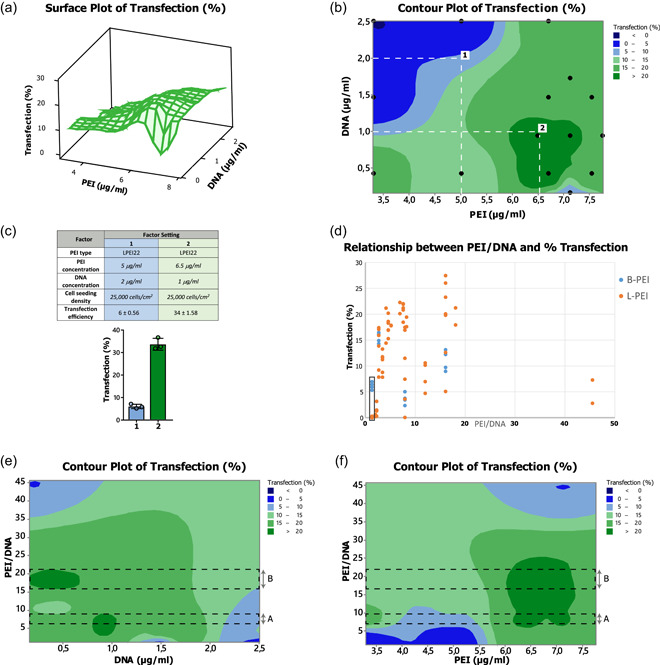
Design of transfections (DoT) model. (a) Surface and (b) contour plots modeling the transfection output in response to PEI and DNA concentration. Black dots in (b) correspond to the points of the designs analyzed, whereas (1) and (2) numbers indicate the combinations of DNA and PEI concentrations tested. (c) Transfection efficiencies obtained in the suboptimal (1) and optimal (2) conditions. (d) Transfection efficiencies obtained at the different PEI/DNA ratios using LPEI (orange) or BPEI (blue) as reagent. The rectangle indicates the PEI/DNA ratio of 1.32, at which BPEI is more efficient than LPEI. (e,f) Contour plots showing transfection efficiency (*Z*‐axis) with respect to DNA (e) or PEI (f) concentration (*X*‐axis) and PEI/DNA ratio (*Y*‐axis). Dashed lines identify two intervals of DNA/PEI ratio (a,b) delimitating the areas of highest values of transfection efficiency (dark green) for both DNA and PEI concentrations

By combining the output data obtained from full fractional, BB and CC designs, keeping constant the optimized levels of PEI type and cell density, a final model was obtained, that we named the DoT model (Figure 5). The DoT model is able to predict the transfection output at different PEI and DNA concentrations and to clearly identify a response surface peak corresponding to the optimized transfection setting. The resulting response surface plot (Figure 5a) and, even more so, the contour plot (Figure 5b) highlighted the best setting of PEI and DNA concentration, able to maximize the transfection efficiency. Finally, to validate the DoT model, we tested two different factor settings: (1) a suboptimal one (5 μg/ml LPEI22, 2 μg/ml DNA, 25,000 cells/cm^2^), corresponding to the amount of DNA and PEI set in the transfection protocol optimized for primary tissue‐derived cells, but using BPEI polymer (Ming Hsu & UludaĞ, 2012), and (2) the optimized conditions here identified (6.5 μg/ml LPEI22, 1 μg/ml DNA, 25,000 cells/cm^2^). For each factor setting, three independent experiments were performed and the transfection efficiencies were measured (Figure 5c). It is worth noting that using the optimized factor setting, we obtained a quite satisfactory MTE of 34%, whereas the suboptimal setting resulted in a mean efficiency of about 6%. In both cases, we obtained the output value predicted by the DoT model (Figure 5b), confirming its validity. These results clearly show the effectiveness of the DoT method in optimizing transfection efficiency.

We also took into account the PEI/DNA ratio, which refers to the balance between PEI negative charges and DNA positive ones, a key factor in transfection. The relationship between the transfection efficiencies obtained in the conditions tested in all the three experimental designs and their relative PEI/DNA ratios is extremely variable (Figure 5d). However, the best outputs at the different ratios are usually obtained by using as transfectant the LPEI molecule, with the exception where the PEI/DNA ratio is close to 1 (1.32), for which BPEI is preferable to LPEI. This is probably due to the higher number of negative charges on the branched conformation, which causes, albeit minimally, an excess of positive charges that seems to favor transfection at this low PEI/DNA ratios. Drawing the contour plots for transfection efficiency vs. PEI/DNA ratio and DNA or PEI, respectively, we highlighted two ranges of PEI/DNA ratio, named A and B, predicting the highest transfection output (Figure 5e,f). The amount of transfectant used per μg of DNA obviously has also economic implications, therefore, lower values, corresponding to the A interval, are preferable, especially when an expensive reagent is used. The optimal conditions identified and tested, that is 1 μg/ml of DNA and 6.5 μg/ml of PEI, correspond to a value of 6.5 for the PEI/DNA ratio, which approximately matches the lower limit of the A interval, also allowing transfection reagent costs to be minimized.

## 4 CONCLUSIONS

**Figure 6:**
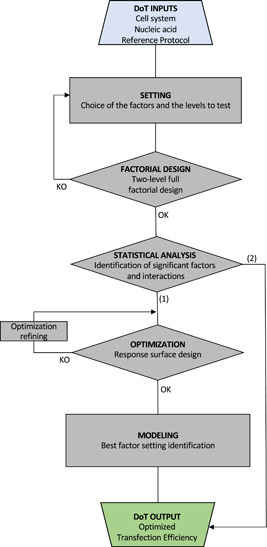
Design of transfections (DoT) workflow. Rectangles represent processes whereas rhombuses represent flow checkpoints. OK indicates that it is possible to continue to the next step, whereas KO indicates that the checkpoint has not been overcome and the step indicated by the arrow has to be repeated. Along Path (1), an OPTIMIZATION stage, through response surface designs, allows to model transfection and, then, to identify the best factor setting that optimizes transfection efficiency (OUTPUT). Optimization can be refined until a good model describing the factor space is obtained. In some cases, the factorial design and the subsequent statistical analysis might be sufficient to identify a satisfactory output (Path 2)

In our DoT assay, we analyzed the impact on neural progenitor transfection of four independent factors, namely PEI concentration, PEI type, DNA concentration, and cell density. Our results highlight that: (i) LPEI is associated to higher transfection efficiency than BPEI; (ii) DNA concentration and especially PEI concentration levels strongly influence transfection efficiency, and, related to this, (iii) the PEI/DNA ratio used for transfection is a good indicator of the obtainable efficiency. Finally, we identified an optimized setting of transfection conditions for neural progenitors (6.5 μg/ml LPEI22, 1 μg/ml DNA, 25,000 cells/cm^2^), able to increase the performance of the transfection assay to 34% of efficiency. The conditions identified could serve as reference for transfection of cells of neural origin, or more generally, cells difficult to transfect, using a simple, safe, and economic reagent such as PEI. Our successful experience in customizing DoE methodology to a specific biological process such as cell transfection allowed us to design and validate a flexible operational flow describing our DoT approach (Figure 6), which can be complemented, if needed, with cost optimization. The formalization of an operational flow together with the detailed presentation and discussion of the data might support in spreading DoE application for transfection optimization as well as for optimization of other biological processes, increasing the scientific critical mass and its impact on different bioprocesses.

## Supporting information

Supporting information.

Supporting information.

Supporting information.

Supporting information.

Supporting information.

## Data Availability

The data that support the findings of this study are available in the supplementary material of this article. Additional data are available from the corresponding author upon reasonable request.
