# Supplementary material for: Design of transfections: Implementation of design of experiments for cell transfection fine tuning
Source: Biotechnol Bioeng. 2021 Sep 1;118(11):4488–502. doi: 10.1002/bit.27918 (PMC9291525; doi:10.1002/bit.27918)

A

StdOrder	RunOrder	CenterPt	Blocks	DNA (µg/ml)	PEI (µg/ml)	Cell density (cell/cm2)	Transfection (%)
7	1	2	1	0.417	5	50000	4.71
3	2	2	1	0.417	6.7	37500	5.06
10	3	2	1	1.46	6.7	25000	20.18
1	4	2	1	0.417	3.3	37500	3.59
15	5	0	1	1.46	5	37500	11.22
11	6	2	1	1.46	3.3	50000	0.30
2	7	2	1	2.5	3.3	37500	0.04
9	8	2	1	1.46	3.3	25000	1.31
4	9	2	1	2.5	6.7	37500	9.21
13	10	0	1	1.46	5	37500	7.83
6	11	2	1	2.5	5	25000	0.48
14	12	0	1	1.46	5	37500	8.76
12	13	2	1	1.46	6.7	50000	16.90
5	14	2	1	0.417	5	25000	10.58
8	15	2	1	2.5	5	50000	0.27
22	16	2	2	0.417	5	50000	6.97
18	17	2	2	0.417	6.7	37500	12.61
26	18	2	2	1.46	3.3	50000	1.47
24	19	2	2	1.46	3.3	25000	3.16
23	20	2	2	2.5	5	50000	0.21
29	21	0	2	1.46	5	37500	13.26
28	22	0	2	1.46	5	37500	11.61
30	23	0	2	1.46	5	37500	11.76
27	24	2	2	1.46	6.7	50000	15.28
16	25	2	2	0.417	3.3	37500	0.05
19	26	2	2	2.5	6.7	37500	7.85
20	27	2	2	0.417	5	25000	10.14
17	28	2	2	2.5	3.3	37500	0.00
21	29	2	2	2.5	5	25000	0.61
25	30	2	2	1.46	6.7	25000	18.59

B

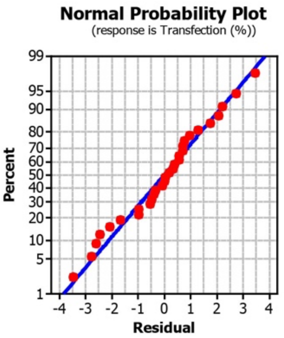

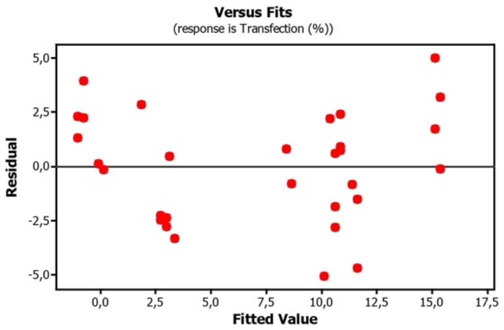

Supplement: Supplementary file 1 — Supporting information. [file BIT-118-4488-s001.pdf]
