# Supplementary figures and images for: Design of transfections: Implementation of design of experiments for cell transfection fine tuning
Source: Biotechnol Bioeng. 2021 Sep 1;118(11):4488–502. doi: 10.1002/bit.27918 (PMC9291525; doi:10.1002/bit.27918)

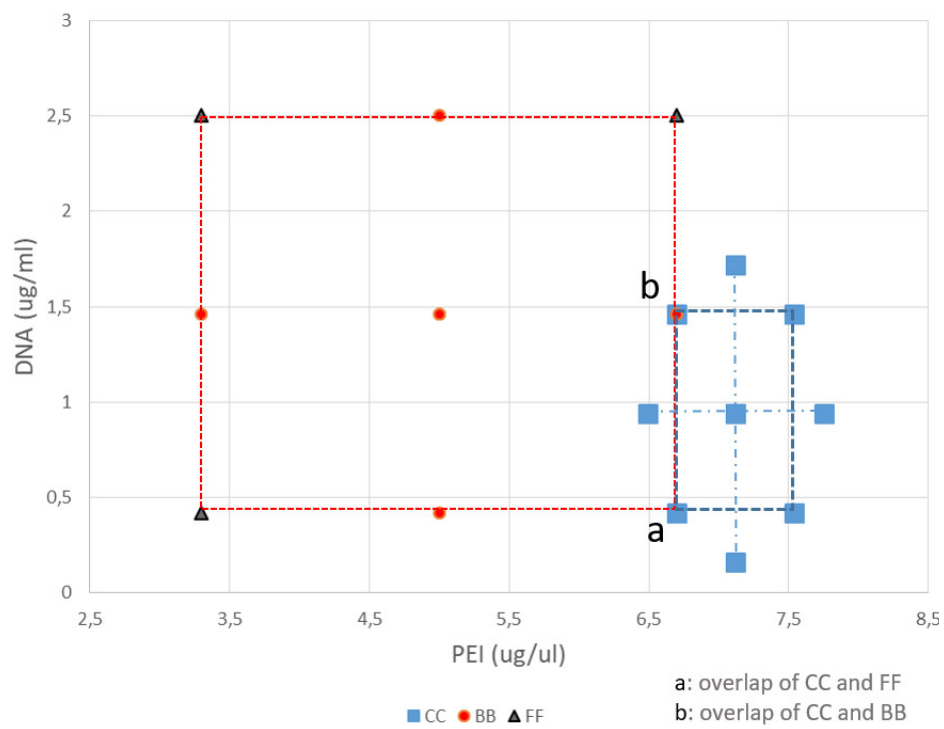

Supplement: Supplementary file 2 — Supporting information. [file BIT-118-4488-s004.pdf]

A

| StdOrder | RunOrder | PtType | Blocks | DNA<br>( $\mu\text{g/ml}$ ) | PEI linear<br>( $\mu\text{g/ml}$ ) | Transfection<br>(%) |
|----------|----------|--------|--------|-----------------------------|------------------------------------|---------------------|
| 16       | 1        | -1     | 2      | 0.1564                      | 7.1187                             | 7.29                |
| 15       | 2        | 1      | 2      | 1.46                        | 7.5375                             | 17.78               |
| 22       | 3        | 0      | 2      | 0.9385                      | 7.1187                             | 21.17               |
| 20       | 4        | 0      | 2      | 0.9385                      | 7.1187                             | 18.44               |
| 21       | 5        | 0      | 2      | 0.9385                      | 7.1187                             | 21.92               |
| 13       | 6        | 1      | 2      | 1.46                        | 6.7                                | 18.27               |
| 18       | 7        | -1     | 2      | 0.9385                      | 6.4906                             | 22.27               |
| 12       | 8        | 1      | 2      | 0.417                       | 6.7                                | 23.28               |
| 17       | 9        | -1     | 2      | 1.7207                      | 7.1187                             | 21.17               |
| 14       | 10       | 1      | 2      | 0.417                       | 7.5375                             | 17.93               |
| 19       | 11       | -1     | 2      | 0.9385                      | 7.7468                             | 17.58               |
| 4        | 12       | 1      | 1      | 1.46                        | 7.5375                             | 17.84               |
| 2        | 13       | 1      | 1      | 1.46                        | 6.7                                | 16.95               |
| 11       | 14       | 0      | 1      | 0.9385                      | 7.1187                             | 22.01               |
| 5        | 15       | -1     | 1      | 0.1564                      | 7.1187                             | 2.78                |
| 6        | 16       | -1     | 1      | 1.7207                      | 7.1187                             | 14.41               |
| 7        | 17       | -1     | 1      | 0.9385                      | 6.4906                             | 20.11               |
| 9        | 18       | 0      | 1      | 0.9385                      | 7.1187                             | 21.20               |
| 10       | 19       | 0      | 1      | 0.9385                      | 7.1187                             | 20.82               |
| 1        | 20       | 1      | 1      | 0.417                       | 6.7                                | 19.80               |
| 3        | 21       | 1      | 1      | 0.417                       | 7.5375                             | 21.22               |
| 8        | 22       | -1     | 1      | 0.9385                      | 7.7468                             | 19.43               |

B

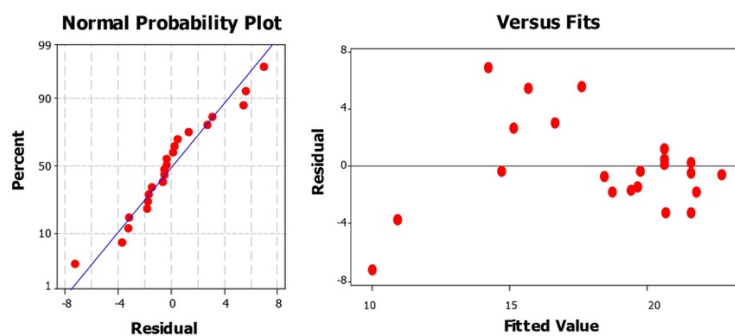

Supplement: Supplementary file 3 — Supporting information. [file BIT-118-4488-s005.pdf]
