# Supplementary material for: Design of transfections: Implementation of design of experiments for cell transfection fine tuning
Source: Biotechnol Bioeng. 2021 Sep 1;118(11):4488–502. doi: 10.1002/bit.27918 (PMC9291525; doi:10.1002/bit.27918)
Supplement: Supplementary file 4 — Supporting information. [file BIT-118-4488-s002.docx]

**Supplementary Table**

Worksheet of the screening two-level full factorial design with all the combinations to test.


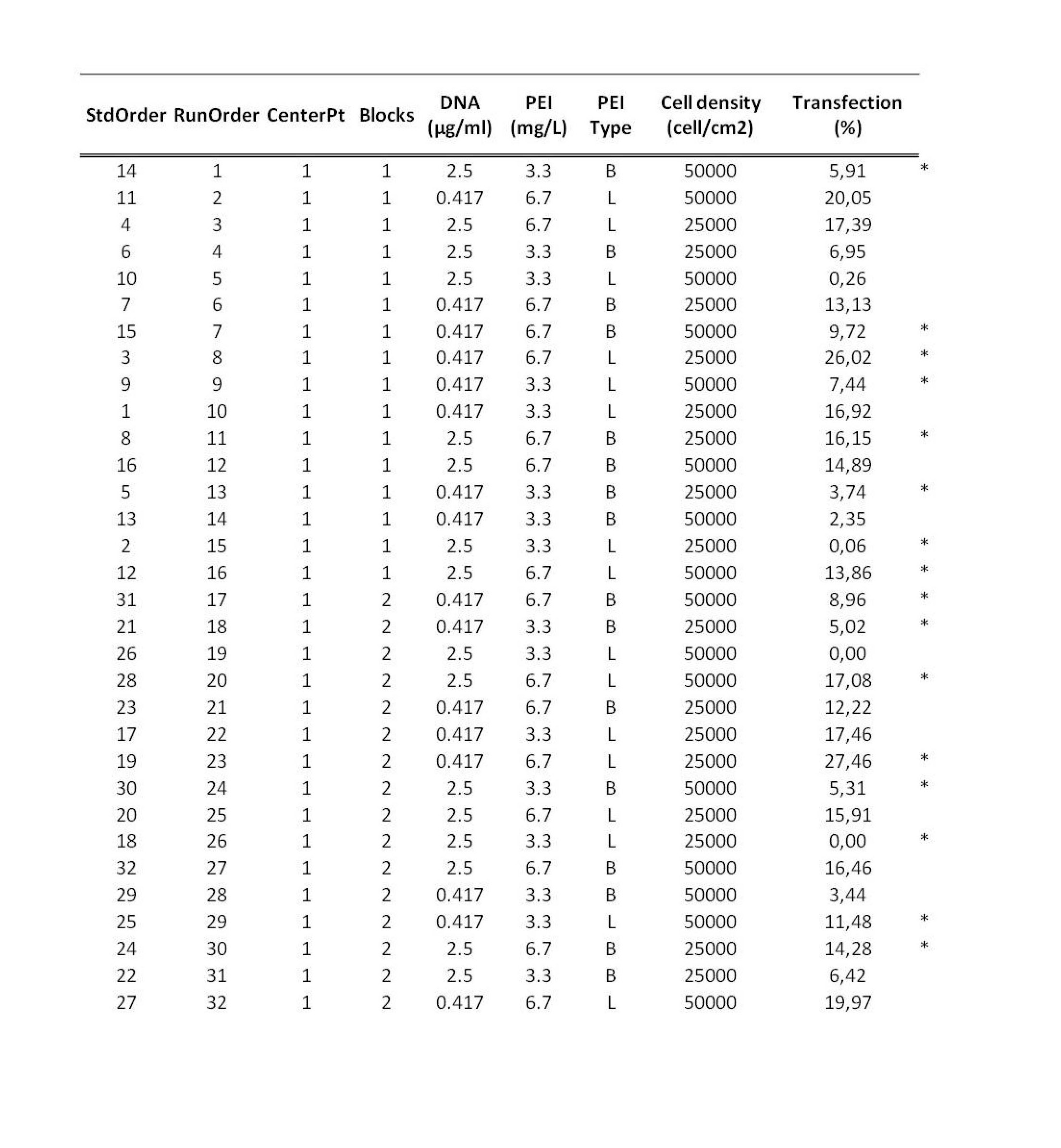


f the Screening Design with all the combination to test

Worksheet of the Screening Design with all the combination to test
