# Supplementary material for: Design of transfections: Implementation of design of experiments for cell transfection fine tuning
Source: Biotechnol Bioeng. 2021 Sep 1;118(11):4488–502. doi: 10.1002/bit.27918 (PMC9291525; doi:10.1002/bit.27918)
Supplement: Supplementary file 5 — Supporting information. [file BIT-118-4488-s003.docx]

**Supplementary Figure Legends**

**Supplementary Figure 1**. **Box-Behnken design.** (A) Worksheet with all the combinations analysed and their relative output. (B) On the left the normal probability plot showing a normal residual distribution, on the right the “residual versus fits” plot showing constant variance of the residuals.

**Supplementary Figure 2. Comparison of the factor space explored by the full factorial (FF), Box-Behnken (BB) and central composite (CC) designs.**

**Supplementary Figure 3**. **Central composite design.** (A) Worksheet with all the combinations tested and the relative output. (B) On the left the normal probability plot showing a normal residual distribution, on the right the “residual versus fits” plot showing constant variance of the residuals.
